# Emerging trends of Zika apprehension in an epidemic setting

**DOI:** 10.1371/journal.pntd.0006167

**Published:** 2018-01-25

**Authors:** Camille Fritzell, Jocelyn Raude, Mirdad Kazanji, Claude Flamand

**Affiliations:** 1 Pasteur Institute of French Guiana, Cayenne, France; 2 UMR “Emergence des Pathologies Virales” (Université Aix-Marseille, IRD 190, INSERM 1207, EHESP), Marseille, France; 3 UMR “Processus Infectieux en Milieu Insulaire Tropical” (INSERM 1187, CNRS 9192, IRD 249, Université de La Réunion), Réunion, France; Evidence Action, AUSTRALIA

## Abstract

**Background:**

French Guiana is a territory that has a decades-long history of dengue outbreaks and more recently, in 2014, a chikungunya outbreak. Zika virus (ZIKV) emerged in late 2015 and subsequently led to an important outbreak.

**Methodology/Principal findings:**

A cross-sectional phone survey was conducted among the general population during the outbreak in June 2016 with a total of 1,129 individuals interviewed to assess perceptions, knowledge and behaviors regarding zika infection. The population seemed aware of zika, and perceived the infection as a more serious health threat than other common mosquito-borne diseases. Furthermore, both the perceptions and behaviors related to zika and its prevention were found to vary considerably among different social groups, geographic areas and gender; less educated female participants were found to perceive the disease as more worrisome and were less likely to adopt protective behaviors. Moreover, female population has been particularly responsive to awareness campaigns and rapidly understood the extent of risks associated with ZIKV infection.

**Conclusions/Significance:**

These results revealed that ZIKV appeared at the time of the survey as a new health threat that concerns the public more than chikungunya and dengue fever with differences observed among subgroups of population. These results have implications for the development of multifaceted infection control programs, including strategies for prevention and awareness, helping the population to develop an accurate perception of the threat they are facing and encouraging behavior changes.

## Author summary

Although dengue fever has been a focus of many awareness campaigns in Latin America, very little information is available about beliefs, attitudes and behaviors regarding vector-borne diseases among the population of French Guiana. Following the end of the first chikungunya outbreak and at the initial onset of the first zika outbreak, a quantitative survey was conducted among 1129 individuals aiming to study the emotional, cognitive and behavioral response to the risk of zika infection and assess variations among different groups of population. People from French Guiana were found to perceive zika substantially differently from other Aedes mosquito-borne diseases. Overall, ZIKV appeared at the time of the survey as a new health threat that makes the population more scared than chikungunya and dengue fever. Furthermore, both the beliefs and behaviors related to zika and its prevention were found to vary considerably among different social groups, gender and geographic areas. Education had an impact on perceptions and behaviors among women. Female population has been particularly responsive to awareness campaigns and rapidly understood the extent of risks associated with ZIKV infection. Overall, findings emphasize the importance of developing appropriate and relevant strategies helping population to engage in protective behaviors adapted to the health threat they are facing. Given the importance of the public response and precautionary actions to control the spread of an emergent threat, additional research on risk perceptions and other behavioral determinants is warranted.

## Introduction

Zika virus (ZIKV) is a Flavivirus transmitted by mosquitoes, primarily *Aedes aegypti*, the same vector that transmits dengue, chikungunya and yellow fever. During the next half century, ZIKV was considered as an emergent virus with few sporadic and imported cases reported in Africa and Asia until 2007, when a major epidemic occurred in Micronesia (Yap)[1]. A second important epidemic occurred in 2013 in the Pacific, affecting French Polynesia [2].

Since its introduction in the Americas in early 2015 in Brazil, ZIKV has rapidly spread through the continent [3,4]. Although ZIKV infections have not historically been considered as a significant public health concern, during this recent emergence, the virus has been linked to neurological disorders and severe congenital abnormalities. Several recent studies have also highlighted that ZIKV can be transmitted through sexual contact or from mother to fetus [5–7]. Today, it became the first major infectious disease linked to human birth defects, putting at risk pregnant women and women of childbearing age [8–10]. To support national governments and communities in preventing and managing the complications of ZIKV, the WHO launched a global strategic response framework for zika in February 2016. One of its objectives was prevention of health risks associated with ZIKV infection through integrated vector management (IVM) [11]. However, human behavior plays a considerable role in the success or failure of prevention programs and policies implemented by the public health authorities to control the spread of the disease. Since the WHO report about the “behavioral and social aspects of malaria and its control,” human behaviors–and their sociocultural determinants–are increasingly recognized as critical factors contributing to risk for and prevention of mosquito-borne diseases. Furthermore, it has been well-established in the literature that health-protective behaviors are strongly guided by a range of mental representations that individuals and communities develop about health threats over time [12–16]. This construct refers to personal conceptions, beliefs, schemata or imagery about an illness and its diverse consequences. To date, the mental representations of mosquito-borne diseases, and particularly ZIKV infection, have received little attention from the global scientific community. Therefore, the objective of this study was to examine public perceptions associated with this new health threat, with the purpose of informing ongoing intervention practices. More specifically, we wanted to determine (1) whether ZIKV infection is perceived differently from other common mosquito-borne diseases, (2) whether a feeling of emerging infectious diseases “fatigue” can be observed in the French Guiana populations, and (3) whether there existed significant variations in the perception of this emerging health threat among population subgroups, particularly between men and women given the unusual transmission routes and potentially dramatic consequences of ZIKV infection.

### Setting

**Fig 1:**
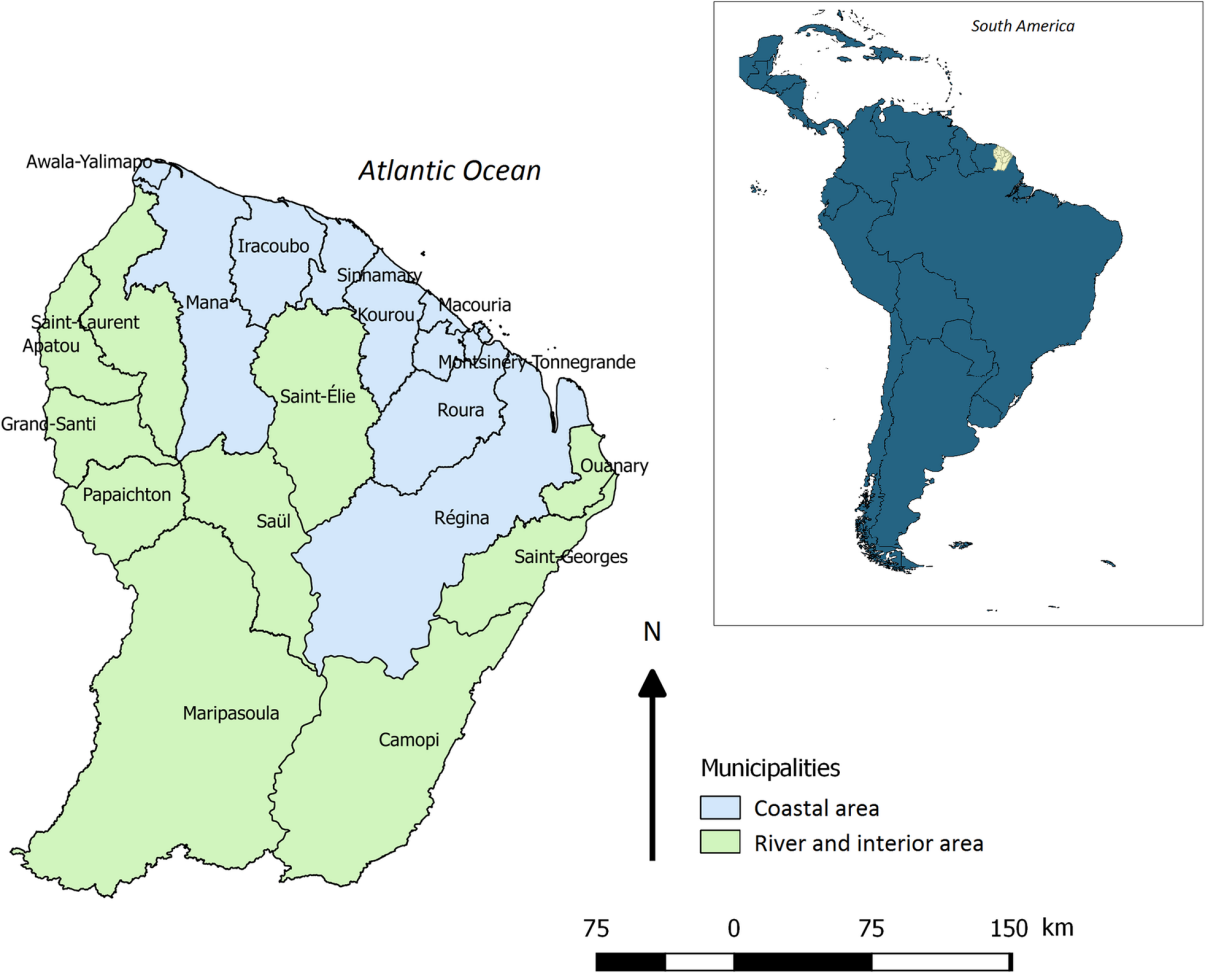
Map of French Guiana, South America, datasource: OpenStreetMap; QGIS 2.18 software.

French Guiana, an overseas region and department of France located in the Amazonian forest complex, is composed of two main inhabited geographical regions: a central, urbanized area including a coastal strip along the Atlantic Ocean, where a large part of the population lives, and a more remote area along the Surinamese and Brazilian frontiers called the “interior area” (Fig 1).

In French Guiana, *Ae*. *aegypti* has been responsible for several major dengue fever outbreaks over the past few decades and a recent outbreak of chikungunya [17,18]. The emergence of ZIKV has been considered of particular concern because the territory has the highest fertility rate in the Americas (3.5 children per woman), with an infant mortality rate (1.2%) that is three times higher than in metropolitan France (0.4%) [19]. On the 22^nd^ of January in 2016, local health authorities launched an official epidemic alert following the rapid spread of ZIKV in the most inhabited part of the territory [20]. The public health response to the outbreak of ZIKV disease included distribution of information about the importance of mosquito bite prevention and the use of physician services for pregnant women with potential symptoms, as well as recommendations by health authorities to delay pregnancy. A massive media campaign was conducted during the outbreak including television and radio spots, prospectus, posters (displayed on the roads and in medical centers) and also newspapers. Messages were about how to prevent mosquito bites or with all zika symptoms listed and there was also specific prospectus for pregnant women. Moreover, all pregnant women were invited to be carefully monitored with a blood sample collected at each trimester of pregnancy then analyzed at the Arbovirus Reference Center at Pasteur Institute of French Guiana [21].

## Methods

### Participants, design and ethical considerations

A cross-sectional phone survey about “beliefs, attitudes and practices” among the general population of French Guiana was conducted from June 15–30 in 2016. Eligibility criteria included (i) having a landline or mobile phone, (ii) being at least 18 years old and (iii) indicating consent for participation.

The sample design was based on a random 2-stage selection procedure, stratified according to the type of phone (50% mobile and 50% land line), municipalities and age. Households were randomly selected at the first stage then one participant was randomly selected among adults living in the selected household. The sample calculation included investigation of 1,100 individuals. The survey was conducted by Ipsos, a French consulting firm that used the CATI system (Computer Assisted Telephone Interviews) and CONVERSO software. To reach the largest possible portion of the population, interviews were carried out from Monday to Friday between 10 am and 8 pm and on Saturday between 10 am and 5 pm.

According to the French legislation, the survey protocol and processing of data collection was subject to a declaration to CNIL, the French National Agency responsible for ethical issues and protection of individual data collection under no. 2043940. All the information was collected anonymously, and the participants were informed of their rights to access and rectify personal information.

### Questionnaire and measurements

Data were recorded using a standardized questionnaire administered by phone.

Aside from socio-demographic variables, collected data were grouped into four general categories: 1) environmental variables and exposure to mosquito, 2) perceptions of the illness and risk of contracting ZIKV infection, 3) perceptions and practices of protective behaviors promoted by the public health authorities to control the spread of the disease and 4) self-reported frequency of protective behavior in response to the zika epidemic.

### Environmental and exposure variables

The questionnaire covered of a wide range of topics such as the type of housing, the presence or absence of potential breeding sites, and potential factors associated with breeding sites. Respondents were also asked how frequently they were bitten by mosquitoes (response options: ‘Never’, ‘Seldom’, ‘Sometimes’ or ‘Often’). Participants were also asked whether they were educated about “*Aedes* mosquitoes”, how frequently they practiced outdoor activities, and during which time of the day mosquito bites occurred. Participants were asked to report the occurrence of an acute febrile illness consistent with presumptive ZIKV infection during the outbreak, and if they had dengue and/or chikungunya virus infection. If the answer was “YES”, we asked them if they had consulted a doctor.

### Cognitive and emotional variables

A broad range of personal beliefs was investigated, especially regarding perceptions of the health threat, i.e., qualitative judgments (based on closed-end questions using unordered response options), and quantitative judgments (based on questions using a Likert response scale with a numerical value ranging from 0 to 10) that individuals expressed when asked to evaluate a specific illness and the risk of contracting it [22]. To characterize these perceptions within the population, questions were drawn from the existing methodological literature using the Brief Illness Perception Questionnaire (B-IPQ) [23]. This questionnaire measures six components: *the identity*- the symptoms that patients associate with the illness; *the cause*- the personal ideas about etiology; *the timeline*- the perceived duration of the illness; *the consequences-* the expected effects and outcome; and *the treatment control*- the effectiveness of treatment methods for recovery from the illness; and the *perceived coherence*- whether people think the threat is easy to understand. Complementary questions were adapted from the risk perception literature devoted to transmissible infectious diseases. Worry, perceived severity, perceived exposure, and perceived susceptibility to the disease were also assessed to explore the multiple components of risk perception [24]. Except for the perceived cause and identity of the disease, respondents were asked to use an 11-point Likert response scale ranging from 0 to 10 to characterize their mental and emotional representations of the health threat associated with a variety of vector-borne diseases.

### Behavioral variables

In line with the health beliefs model [25], participants were asked how often they practiced health-protective behaviors as they relate to various vector control methods (e.g., wearing long-sleeved clothing or using repellent) and vector control measures (e.g., covering water receptacles). Participants were then asked whether those behavioral recommendations were effective in preventing mosquito bites (response options: ‘Very ineffective’, ‘Somewhat ineffective’, ‘Somewhat effective’, ‘Very effective’ and ‘Unsure’). To obtain a mean protection score for each respondent, a level of protection score was created as the sum of each mean used.

### Data analysis

Statistical analyses were performed using STATA12 software (Stata Corp., College Station, TX, USA) [26] and SPAD8 [27]. Primary sampling units and strata were considered for calculating estimations according to the design effect. Post-stratification weights were used to correct potential biases due to misrepresentation of demographic characteristics, including gender and education level. All estimations were obtained using STATA12 “svy” commands.

Bivariate analyses were conducted using Chi-square tests to compare proportions, and linear combinations tests and regression combined with Wald tests were used to compare mean scores of risk perceptions. The level of statistical significance was set to (p = 0.05). Data were adjusted according to gender and level of education.

In addition, a multiple correspondence analysis and a hierarchical cluster analysis were performed to determine the natural groupings of observations regarding the level of knowledge about zika disease and its issues in order to cluster the population in different groups according to their literacy.

South America and French Guiana layers were drawn using data from OpenStreetMaps (http://www.openstreetmap.org) and mapping operations have been done using QGIS 2.18 software [28].

## Results

There was a total of 1,129 participants to the survey. The mean age of participants was 45.6 years old ranging from 18 to 96 years. The original sample showed over-representation of women (64.8% vs 50% in the general population of French Guiana) and well-educated people (38% vs 28.2% in French Guiana). All the results presented later in this article were restated to reflect the post-stratification adjustment.

### Knowledge, beliefs and behaviors

Over half of the participants reported having knowledge of *Aedes* mosquitoes (59.1%, 95% Confidence Interval (CI): 54.8–63.3), and 87.9% (95% CI: 84.7–90.5) properly identified zika as a vector-borne disease. The most commonly mentioned symptoms associated with zika disease were fever and myalgia (91%, 95% CI: 87.9–93.4 and 60%, 95% CI: 55.7–64.2, respectively), followed by headache and arthralgia (49.2%, 95% CI: 44.9–53.4 and 44.8%, 95% CI: 40.6–49, respectively). Symptoms mentioned the least frequently were conjunctivitis (8.5%, 95% CI: 6.4–11) and neurologic disorders (1.6%, 95% CI: 0.9–2.8). Most participants were aware of zika transmission issues: 79.3% (95% CI: 75.6–82.6) knew that ZIKV can be transmitted from mother to child, 55.7% (95% CI: 51.5–59.9) knew that ZIKV can be sexually transmitted. Finally, 54.4% (95% CI: 50.1–58.6) declared to be well-informed about zika disease. However, 17.3% (95% CI: 14.-20.9) reported that there was a vaccine against zika. The most popular sources of information about the disease were television, radio and posters (85%, 95% CI: 81.4–87.9, 66%, 95% CI: 61.9–70 and 63.1%, 95% CI: 58.8–67.2, respectively). The proportions of respondents claiming to have been previously infected with zika, chikungunya or dengue were of 8.8% (95% CI: 6.8–11.5), 14.3% (95% CI: 11.4–17) and 20.7% (95% CI: 17.7–24.1), respectively. Among those who reported sudden onset of high fever during the zika outbreak (10.5%, 95% CI: 88–17), 83.8% claimed to have seen a doctor. The WHO recommended a number of IVM actions for individuals to prevent mosquito bites and thus the spread of the virus, thereby reducing their own personal risk. Among respondents who declared being bitten by mosquitoes either often or every day, 40.5% (95% CI: 36.4–44.6) and 66.5% (95% CI: 60.1–72.3), respectively reported taking preventive measures. When asked about the effectiveness of several preventative measures, the most commonly reported were emptying of water from receptacles, use of bed nets, and covering storage containers, whereas the less effective measures were extensive insecticide spraying and the use of a vaporizer for outdoor insecticides. The most frequently reported preventive measures were closing the door and reducing outdoor activities (47.7%, 95% CI: 43.4–52) and using window nets (32.43%, 95% CI: 28.7–36.4). Overall, almost 20% (95% CI: 15.7–22.1) of the population took at least 5 actions to prevent mosquito bites.

### Differences between perception of zika and other vector-borne diseases

**Fig 2:**
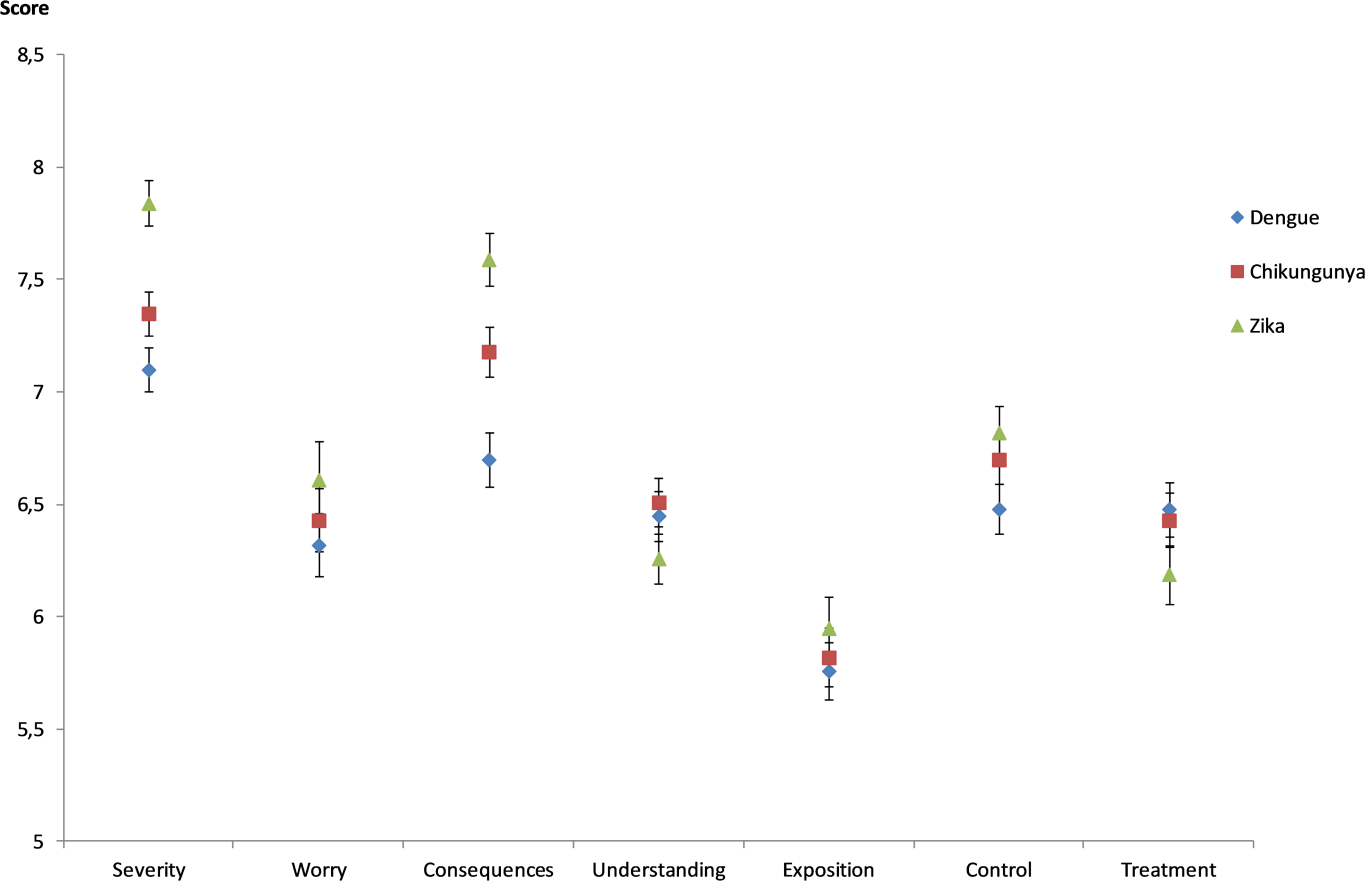
Mean threat perception scores regarding dengue, chikungunya andz ika infections in French Guiana, 2016.

As shown in Fig 2, zika received a significantly higher mean score than did dengue and chikungunya as a response to questions about worry, perceived severity, perceived consequences and perceived exposure (p<0.01). Regarding zika treatment, its perceived efficiency was significantly lower than dengue and chikungunya treatments (p<0.01). Moreover, zika appeared to be less understood than chikungunya disease (p<0.01). Nonetheless, ZIKV infection was perceived as more easily avoidable than dengue (p = <0.01).

### Differences in perception and attitudes about zika disease among populations

The distribution of risk perceptions and behaviors among several subpopulations are presented in Table 1. Analysis revealed an association between age, perceived worry, level of understanding, and behaviors, both with a gradient in responses. Respondents between 18 and 25 years old were more worried about zika (p = 0.016), more likely to adopt protective measures (p<0.01), and they claimed to understand the disease less compared to claims of older age groups (p = 0.047). Individuals living in the interior were less worried about zika than those living in the coastal area (p<0.01) and were more likely to adopt protective measures (p = 0.019).

**Table 1:** Risk perceptions and behaviors associated with zika disease among several subpopulations, French Guiana, 2016.

Respondents characteristic	Mean score of perceptions and protection associated with zika																	
	Severity	p-value	Worry	p-value	Consequences	p-value	Understanding	p-value	Exposition	p-value	Control	p-value	Treatment	p-value	Risk of infection	p-value	Behaviors	p-value
**Gender**																		
*Female*	8,27	<0.001	7,12	<0,001	8,05	<0,001	6,45	0,166	6,41	<0,010	7,02	0,076	6,34	0,257	6,62	0,022	2,51	0,330
*Male*	7,39		6,08		7,12		6,08		5,47		6,61		6,05		6,08		2,35	
**Age**																		
*[18–25]*	8,02	0,539	7,40	0,016	7,66	0,696	5,47	0,047	5,41	0,222	6,46	0,241	6,44	0,502	6,45	0,950	2,96	<0,010
*[26–39]*	7,78		6,81		7,56		6,38		6,14		7,10		6,08		6,38		2,53	
*[40–50]*	7,62		6,41		7,42		6,50		6,05		6,68		5,98		6,29		2,22	
*>50*	7,98		5,86		7,77		6,56		6,05		6,94		6,39		6,29		2,00	
**Zone**																		
*Coastal area*	7,83	0,996	6,83	<0,010	7,62	0,610	6,19	0,204	5,97	0,777	6,91	0,124	6,26	0,390	6,32	0,585	2,33	0,019
*Interior area*	7,84		5,78		7,48		6,56		5,87		6,49		5,98		6,48		2,77	
**History of dengue**																		
*Yes*	7,92	0,230	6,07	0,046	7,31	0,126	7,01	<0,010	6,14	0,414	6,42	0,030	5,41	<0,010	6,41	0,824	2,61	0,235
*No*	7,64		6,78		7,70		6,09		5,88		6,96		6,39		6,35		2,38	
**History of chikungunya**																		
*Yes*	7,59	0,397	6,31	0,438	7,26	0,182	6,11	0,576	6,37	0,157	6,44	0,134	5,59	0,078	6,72	0,192	2,48	0,779
*No*	7,89		6,66		7,68		6,31		5,90		6,92		6,29		6,30		2,41	
**History of zika**																		
*Yes*	7,36	0,158	6 ,00	0,168	7,55	0,842	6,58	0,461	7,47	<0,001	5,89	0,018	5,94	0,520	7,32	<0,010	3,16	<0,010
*No*	7,88		6,67		7,61		6,27		5,79		6,92		6,24		6,24		2,36	
**History of any of three arbovirus**																		
*Yes*	7,54	0,050	6,09	0,010	7,36	0,107	6,54	0,112	6,35	0,017	6,36	<0,010	5,69	<0,010	6,65	0,049	2,64	0,055
*No*	7,99		6,89		7,71		6,12		5,72		7,07		6,48		6,19		2,31	
**Level of education**																		
*Primary school*	7,85	<0,001	6,79	0,080	7,66	<0,001	5,38	<0,010	5,59	0,070	6,52	0,026	6,89	<0,001	6,28	0,546	2,41	0,660
*Some secondary school*	798		6,67		7,74		6,26		6,01		6,99		6,42		6,44		2,40	
*Completed high school*	7,88		6,75		7,64		6,68		5,67		6,96		5,8		6,07		2,38	
*Some college and higher*	7,25		6,13		6,96		6,85		6,28		6,41		5,07		6,34		2,58	

Our results show that previous infection with dengue, chikungunya or zika virus was associated with different perceptions of zika disease. Specifically, individuals reporting previous infection judged the disease less severely (p = 0.05), worried less (p = 0.01), felt more exposed (p = 0.017), had lower estimates for control (p<0.01) and treatment efficiency (p<0.01), and felt more vulnerable in the context of outbreak (p = 0.049).

Finally, respondents with a high level of education characterized the disease as being less severe (p<0.001), as affecting patients to a lesser extent (p<0.001), and as being less avoidable (p = 0.026); highly educated participants also better understood the disease (p<0.01) and judged the treatment as less efficient than respondents with a lower level of education (p<0.001).

As shown in Table 1, women were more afraid of zika than were men. Women were significantly more worried about zika (p<0.001), felt more exposed (p<0.001), and characterized the disease as more severe (p<0.001) and as affecting the patient more than did men (p<0.001). Although scores were higher for women in terms of understanding of the disease and control and treatment efficacy, these differences were not statistically significant. Interestingly, women were worried about disease independently of the level of knowledge, whereas among men, there was an association between knowledge and risk perception (p = 0.03); 65% of men with strong knowledge of the disease were worried vs. 50% among those who were not knowledgeable.

When analyzed according to specific subgroups, women presented nuanced responses with regard to the level of education (Table 2). The impact of education on perceptions was higher among women than men. Low-educated women worried more about the disease (p = 0.02) and were less knowledgeable (p<0.001). Moreover, the level of education was also associated with the adoption of protective behaviors (p<0.01).

**Table 2:** Risk perceptions and behaviors associated with zika disease among women and men according to different subgroups, French Guiana, 2016.

**Respondents characteristic**	**Mean score of perceptions and protection associated with zika among women**																	
	Severity	p-value	Worry	p-value	Consequences	p-value	Understanding	p-value	Exposition	p-value	Control	p-value	Treatment	p-value	Risk of infection	p-value	Behaviors	p-value
**Age**																		
*[18–25]*	8,70	0,119	7,44	0,152	8,28	0,637	5,48	0,067	6,23	0,694	6,98	0,570	6,43	0,601	7,07	0,388	2,73	0,117
*[26–39]*	8,10		7,41		8,00		6,70		6,66		7,21		6,30		6,40		2,78	
*[40–50]*	8,14		7,00		7,88		6,78		6,34		6,7		6,07		6,50		2,26	
*>50*	8,31		6,55		8,09		6,57		6,29		7,13		6,65		6,66		2,12	
**Zone**																		
*Coastal area*	8,30	0,654	7,37	<0,010	8,04	0,980	6,37	0,323	6,46	0,650	7,11	0,247	6,37	0,756	6,65	0,592	2,43	0,127
*Interior area*	8,17		6,23		8,05		6,74		6,24		6,73		6,27		6,48		2,79	
**History of any of three arbovirus**																		
*Yes*	7,94	0,019	6,84	0,168	7,70	0,028	6,86	0,049	6,77	0,057	6,52	<0,010	5,53	<0.001	6,78	0,397	2,65	0,356
*No*	8,45		7,28		8,24		6,22		6,22		7,3		6,81		6,53		2,44	
**Level of education**																		
*Primary school*	8,44	<0.001	7,40	0,020	8,07	<0.001	6,13	0,259	5,84	0,193	6,97	0,220	7,19	<0.001	6,52	0,692	2,39	<0,010
*Some secondary school*	8,46		7,29		8,30		6,37		6,67		7,2		6,68		6,75		2,46	
*Completed high school*	8,16		6,96		7,89		6,79		6,13		6,92		5,76		6,37		2,36	
*Some college and higher*	7,59		6,43		7,36		6,81		6,41		6,92		4,90		6,47		2,88	
**Respondents characteristic**	**Mean score of perceptions and protection associated with zika among men**																	
	Severity	p-value	Worry	p-value	Consequences	p-value	Understanding	p-value	Exposition	p-value	Control	p-value	Treatment	p-value	Risk of infection	p-value	Behaviors	p-value
**Age**																		
*[18–25]*	7,38	0,800	7,370	<0,010	7,06	0,760	5,48	0,430	4,64	0,180	5,97	0,280	6,45	0,670	5,87	0,750	3,15	0,020
*[26–39]*	7,39		6,050		7,01		5,99		5,48		6,96		5,80		6,35		2,23	
*[40–50]*	7,17		5,880		7,03		6,25		5,80		6,67		5,91		6,11		2,19	
*>50*	7,66		5,190		7,44		6,54		5,81		6,74		6,15		5,91		1,89	
**Zone**																		
*Coastal area*	7,37	0,850	6,280	0,100	7,19	0,400	6,01	0,450	5,46	0,990	6,71	0,270	6,16	0,390	5,98	0,300	2,23	0,060
*Interior area*	7,45		5,270		6,81		6,35		5,46		6,22		5,65		6,48		2,75	
**History of any of three arbovirus**																		
*Yes*	7,12	0,310	5,290	0,020	6,98	0,580	6,20	0,640	5,90	0,110	6,19	0,080	5,86	0,510	6,51	0,060	2,63	0,080
*No*	7,52		6,490		7,19		6,01		5,23		6,83		6,15		5,86		2,20	
**Level of education**																		
*Primary school*	7,02	0,080	5,960	0,430	7,06	0,054	4,31	<0,010	5,22	0,120	5,87	0,040	6,48	0,070	5,94	0,720	2,45	0,900
*Some secondary school*	7,55		6,100		7,24		6,15		5,41		6,80		6,19		6,15		2,36	
*Completed high school*	7,59		6,530		7,38		6,57		5,19		7,00		5,85		5,77		2,39	
*Some college and higher*	6,84		5,760		6,48		6,88		6,12		6,16		5,26		6,19		2,21	

Even if the most popular sources of information (television, radio and posters) were reported by the major part of respondents, some variations were observed. Participants aged between 18 and 25 years were more likely to access to social network (p = 0.01) and highly educated participants were more likely to access poster (p = 0.03). Participants who had heard about zika from television (p = 0.010), radio (p<0.001), poster campaign (p<0.001), social network (p = 0.035) and from leaflets (p<0.001) reported to be significantly more informed about zika disease. However, only participants having been aware through leaflets (p = 0.023) were more knowledgeable about zika disease and its issues than those who did not.

Men and women were equally split on the issue of leaving French Guiana in case of pregnancy during a zika outbreak, with 52% and 49% claiming they should have left the territory. Both men and women seemed to be compliant with the recommendations of the WHO, with 85% claiming that they would use a condom during sexual activity. However, approximately 65% of women claimed they would postpone a pregnancy in case of zika outbreak, whereas only 51% of men claimed they would postpone pregnancy. Women indicated that they would worry about undergoing pregnancy during a zika outbreak (75%).

## Discussion

Since its emergence in the Americas in the late 2015, the zika epidemic received considerable attention from the medical, political and lay communities worldwide. During the year 2016, prior to the recent slowdown in transmission of the disease in Latin America, the increasing magnitude of ZIKV outbreaks as well as the severity of their consequences on human health raised the specter of a new and potentially major public health crisis, comparable to those caused by other dramatic re-emerging infectious diseases such as Ebola or SARS [29,30]. Intense public concern was expressed about enhanced transmission of ZIKV which was associated with extensive media coverage, strong institutional attention and high perceptions of health risk [31]. Indeed, since the pioneering works conducted by Slovic & al. in the field of risk psychology [32], it has been well established that diseases caused by invisible, unfamiliar, communicable, potentially highly pathogenic, emerging infectious agents which remains (to a large extent) beyond individual and institutional control are likely to trigger public panic [33].

At both the societal and individual level, one common strategy to cope with unknown and uncontrollable infectious diseases is to relate them to past or existing threats to population health [34]. This social cognitive process, referred to as an “anchoring” effect in social and psychological sciences, consists of actively selecting a small number of familiar events, schemata, images or symbols to describe a new phenomenon. In an epidemic setting, the propensity to use existing or past diseases to understand a new epidemiological phenomenon enables people to make unfamiliar illnesses more familiar, and therefore less threatening [35]. However, it has also been shown that the utilization of some anchors rather than others in the social representation of a new health threat can lead to more or less adaptive responses to the epidemiological event, which may have serious consequences for population health [14,36].

Despite the growing acknowledgment in the scientific community that the cognitive representations held by individuals and groups play a major role in the control and prevention of emerging infectious diseases, little attention has been given to public perceptions of ZIKV infection. To the best of our knowledge, this study is the first to investigate the beliefs, attitudes and practices related to ZIKV among the general population in an epidemic setting. The survey was conducted during the emergence of ZIKV in French Guiana, an endemic region for dengue fever, and nearly one year after a major outbreak of chikungunya, another re-emerging mosquito-borne disease. At the time of the study, the surveillance system estimated a zika prevalence of approximately 3% with 7,830 estimated clinical cases, whereas the study revealed a prevalence of 9%, suggesting an important proportion of clinical cases not covered by monitoring systems.

Our empirical data show that the cognitive representations of ZIKV infection characterizing the French Guiana population were partly anchored on those of other *Aedes* mosquito-borne diseases, specifically chikungunya. Approximately 90% of the respondents were aware that mosquitoes transmit ZIKV, and a large proportion were aware of the specific transmission issues related to the virus, particularly women. For instance, almost 80% of participants claimed that the virus could be transmitted from mother to fetus. This awareness is likely attributable to the massive media coverage of transmission patterns, as well as institutional campaigns about zika control and prevention. However, we note that despite intense media coverage of this issue, only slightly more than a half of the participants knew that ZIKV can be sexually transmitted. In the same vein, only 8.5% and 1.6% correctly identified conjunctivitis and neurologic disorders as common symptom of ZIKV infection. The poor recognition of typical symptoms associated with zika in our study population suggest some anchoring effects, through which clinical manifestations of this disease were confounded with those of other common mosquito-borne diseases such as dengue fever and chikungunya.

Nevertheless, people from French Guiana were found to perceive zika substantially differently from other Aedes mosquito-borne diseases. Specifically, the study shows that zika was associated with higher mean scores in terms of worry, perceived severity, and consequences of infection. Overall, ZIKV appeared at the time of the survey as a new health threat that concerns the public more than chikungunya and dengue fever. Therefore, these results do not give empirical support to the emerging infectious diseases fatigue hypothesis that was developed in the aftermath of the 2009 H1N1 influenza epidemic [34]. According to this hypothesis, the relative apathy of the Western populations regarding health threats related to emerging infectious diseases can be attributed to the increased frequency of alarmist discourse, which is likely to cause some habituation effects. In other words, the cognitive and affective responsiveness of the public to a re-emerging infectious disease does not seem to be impacted by the repetition of warnings, as long as this new health threat is associated with a significant degree of perceived uncertainty about transmission and potential consequences for human health.

Our results also showed that both the perceptions and behaviors related to zika and its prevention vary considerably among different social groups and geographic areas. Notably, there was a social gradient in the cognitive and affective responses to the risk of ZIKV infection; more educated participants were found to perceive the disease as less severe and controllable and more understandable. Some gender effects were also observed during the epidemic, as there were systematic and significant differences in the way men and women perceive the health threat related to the spread of zika in the region. Female population has been particularly responsive to awareness campaigns and rapidly understood the extent of risks associated with ZIKV infection. This result is not surprising, since (1) ZIKV has been found to cause severe fetal anomalies among babies born to infected women, and (2) the ‘gender gap’ is one of the best-documented phenomena of social and cultural influence in the field of risk perception [37,38].

However, results showed that perceptions and behaviors discrepancies appeared among women. While the level of education had a weak impact on risk perception among men, it was clearly identified as a potential determinant of risk perception among women. Importantly, the level of education was also associated with the adoption of protective measures among women. This finding provides health authorities the opportunity to target and adapt future health messages to less advantaged women. Institutions in charge of epidemic and emergent diseases should thus reach this population visiting mother and child protection centers which are numerous in French Guiana both located in the coastal area and in the interior. Actions should first reassure this population about risks associated with zika and emphasize on the importance of protective measures adoption during an outbreak to encourage behavior changes. In fact, a previous study revealed that the level of comprehension was associated with the adoption of protective behaviors [17].

Findings showed that the main communication media were available to all subgroups of population, even less advantaged people had access to television. However, leaflets seemed to be one of the most informative mean and this is not surprising since a lot of information can be transmitted through this medium, whether a television spot must be short, posters must be able to be seen from a distance and no visual data are broadcasted through radio. A continuous effort is thus needed to efficiently transmit prevention messages and target populations through leaflets and maybe adapt others means of communication to be more efficient.

Limitations of the study are inherent to the design of the study which was based on a phone recruitment process. In fact, the interview lasted 25 minutes and may lead to selection biases with more educated and advantaged participants that would have been more sensitive to public health issues addressed by the study. Moreover, the network coverage is heterogeneous on the territory and some uncovered isolated municipalities representing 7 municipalities (out of 22) could not be investigated. However, these failures concerned isolated and rural villages poorly or not affected by the presence of *Ae*. *aegypti*. These areas represent less than 5% of the total population.

### Conclusions

The results of our survey indicated that the public perceived zika disease as a more serious potential health threat than other common mosquito-borne diseases, even though a range of elements within cognitive representations of zika were statistically and graphically found to be anchored on those of chikungunya. The survey helped to identify a subgroup of population shaped by specific risk perceptions and behaviors that deserves further attention given the importance of the public understanding and mental representation of illnesses in the adoption of effective protective behaviors. If this assessment seems difficult to conduct in an epidemic setting, high-risk groups identified may be targeted as a priority in case of a new emergence.

## Supporting information

S1 Checklist STROBE checklist. (DOCX)
